# Vasospastic Angina in a Young Woman: A Case Report

**DOI:** 10.7759/cureus.49640

**Published:** 2023-11-29

**Authors:** Misa Yoshida, Yuichi Orita, Chikage Oshita, Yuko Uchimura, Hiroki Teragawa

**Affiliations:** 1 Department of Clinical Education, JR Hiroshima Hospital, Hiroshima, JPN; 2 Department of Cardiovascular Medicine, JR Hiroshima Hospital, Hiroshima, JPN

**Keywords:** bronchial asthma, vasospastic angina, radial artery spasm, young female, coronary spasm

## Abstract

Vasospastic angina (VSA) is a disease that causes myocardial ischemia due to transient vasoconstriction of the epicardial coronary arteries. This disease generally occurs in middle-aged and older adults, but there are also reports of it occurring in young people. We report a case of VSA in a woman in her 20's. Six months ago, a female patient in her 20s became aware of a strangling sensation in the chest that lasted for approximately 1-20 minutes at rest or during stress. She consulted her family doctor who prescribed nitroglycerin sublingual tablets, which were effective. She was a current smoker and had a history of bronchial asthma, with no family history of coronary artery disease. Resting electrocardiogram and echocardiography revealed no clear abnormalities. The patient was referred to our hospital for coronary angiography (CAG) and spasm provocation test (SPT), primarily to thoroughly examine her chest pain at rest. CAG revealed no significant stenosis. A subsequent SPT using acetylcholine demonstrated diffuse coronary spasm in the left anterior descending coronary artery (LAD). The coronary spasm resolved spontaneously, but the catheter was difficult to maneuver owing to the radial artery spasm at the puncture site; thus, nitroglycerin was administered, which alleviated the radial artery spasm. Another SPT was performed on the right coronary artery (RCA) and revealed no coronary spasm. Coronary microcirculatory function using a pressure wire in response to the peripheral infusion of adenosine triphosphate was assessed in the RCA and LAD, both of which were normal. The patient was discharged from the hospital on an oral calcium channel blocker (CCB). She continued to experience chest pain, but her chest symptoms improved with CCB medication and a change in her workplace. It must be kept in mind that coronary spasms can occur even in young women and should be one of the differentials of chest pain in such patients.

## Introduction

Vasospastic angina (VSA) is a transient, abnormal vasocontraction of epicardial coronary arteries, which run along the surface of the myocardium, leading to myocardial ischemia [1-4]. The prognosis for VSA is generally good [5]; however, acute myocardial infarction and sudden death may occur when the coronary spasm is destabilized [6]. Therefore, diagnosing VSA before treatment is advisable.

VSA, similar to atherosclerotic angina, mostly occurs after middle age [7]. In females, estrogen protects the vasculature, including the coronary artery, from injuries [8], and menopause is considered an important factor in developing VSA in women [7]. Thus, younger patients with VSA are relatively rare [9-11]. However, coronary spasm in young people has recently received more attention [10], and a recent guideline also deals with coronary spasm in children [4]. We experienced a female patient with VSA in her 20s to estimate the disease mechanism. Herein, we report a case of a young female with VSA to reaffirm the importance of VSA in diagnosing chest pain in young females.

## Case presentation

A 28-year-old female patient presented with a chief complaint of chest strangulation at rest or during stress. The patient was aware of chest strangulation that lasted for approximately 10 seconds and was treated medically for suspected reflux esophagitis, which was ineffective. Chest pain attacks occurred several times a week, often several times a day, and lasted from 10 seconds to 15-20 minutes. She had radiating pain in her upper left arm and entire back, with no cold sweat. After using nitroglycerin (NTG) tablets prescribed by the family doctor, the symptoms disappeared within 5 minutes. She was followed by her family doctor, but the frequency of attacks did not improve; therefore, she was referred to our hospital in March 2021 for a thorough examination.

Her medical history included childhood asthma, and she was on medication for bronchial asthma since age 27. Her present medications included montelukast sodium at 10 mg/day, ciclesonide at 400 μg/day, and sublingual NTG at 0.3 mg (for chest pain attacks). A family history of any disease was not noted. As for habits, alcohol intake included Japanese spirit with soda at 350 ml every two days and cigarette consumption at 6 sticks/day for 6 months. Menstrual cycles were mildly irregular but mostly normal. Menstrual cycle and chest symptoms demonstrated no association.

The patient’s height was 153 cm, weight was 49 kg, and body mass index was 20.96 kg/m^2^. Her blood pressure was 136/82 mmHg, pulse rate was 73/min, and oxygen saturation was 99% under room air. Jugular venous distention was not observed. No obvious heart murmur was heard in the chest, and normal alveolar sounds with no rales were heard. The abdomen was flat and soft; the extremities showed no edema.

Her blood biochemistry tests revealed no specific abnormal findings: the level of low-density lipoprotein cholesterol was 86 mg/dL, the level of hemoglobin A1c was 5.6%, the level of C-reactive protein was 0.08 mg/dl, and the level of troponin T was 0.03 ng/mL. Chest radiographs and resting electrocardiogram revealed no abnormal findings. Transthoracic echocardiography demonstrated normal left ventricular wall motion. The peripheral vascular function tests revealed 8.6% and 27.1% flow-mediated dilation (FMD, normal range is >7.0%) and NTG-induced dilation (NID, normal range is considered as >20%) of the brachial artery, respectively, and reactive hyperemia peripheral artery tonometry (RHI-PAT) index was 1.19 (normal range is >2.10).

**Figure 1:**
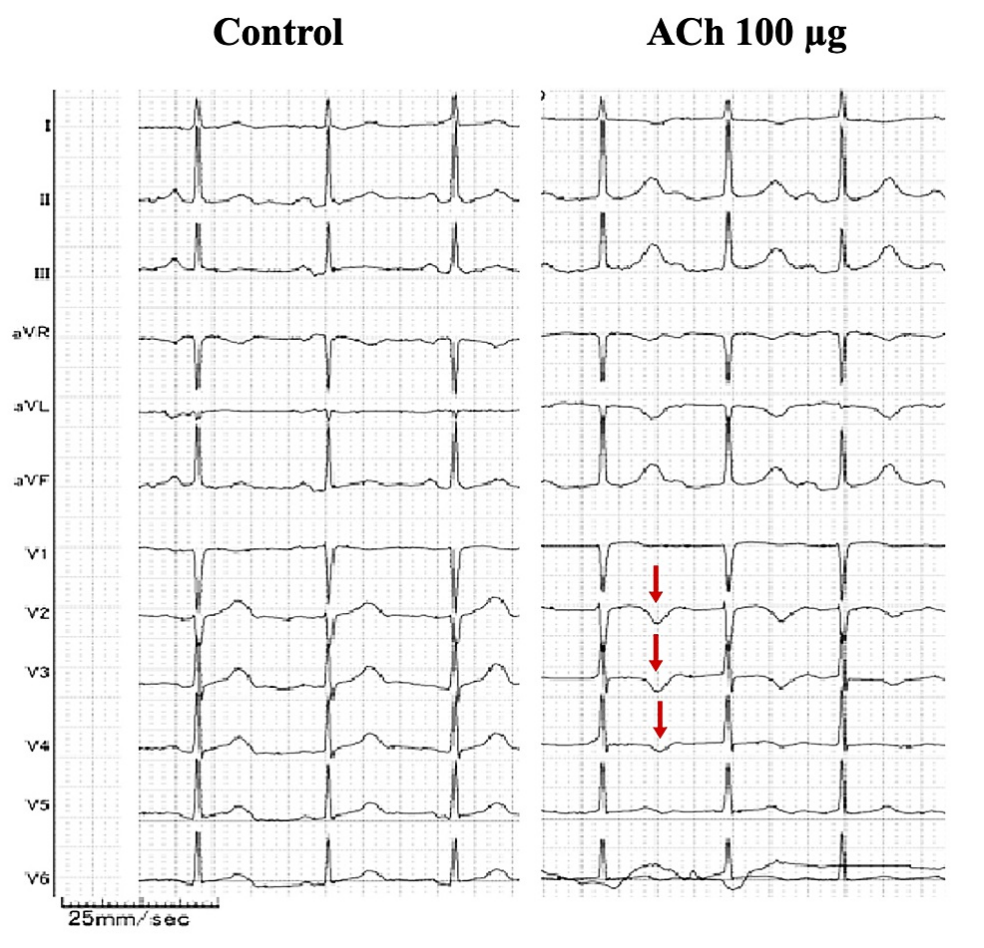
ECGs during the SPT The left panel shows ECG at baseline, and the right panel presents ECG during the SPT using ACh at 100 µg. The changes in T waves during the SPT are indicated by red arrows. ACh: Acetylcholine; ECG: Electrocardiogram; SPT: Spasm provocation test

**Figure 2:**
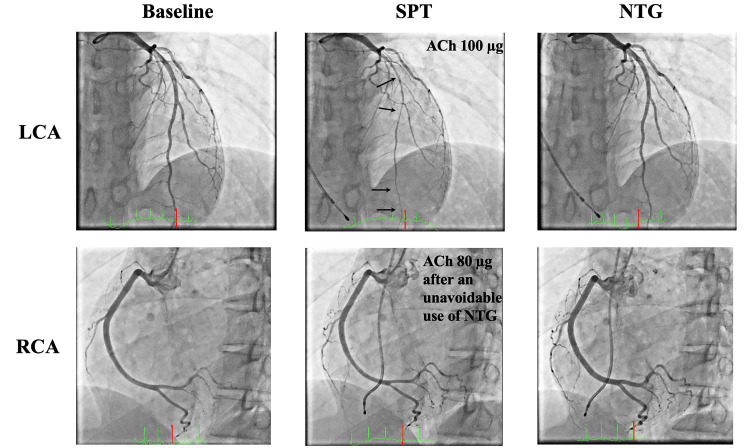
Results of CAG and SPT The upper panels show the coronary angiograms of the LCA at baseline, ACh at 100 µg, and NTG. After administering ACh at 100 µg, a diffuse coronary spasm occurred on the LAD, as indicated by arrows. The lower panels show the coronary angiograms of the RCA at baseline, ACh at 80 µg, and NTG. Coronary spasm was not induced in the RCA by ACh infusions, possibly due to an unavoidable use of NTG to relieve radial artery spasm. ACh: Acetylcholine; CAG: Coronary angiography; LAD: Left anterior descending coronary artery; LCA: Left coronary artery; NTG: Nitroglycerin; RCA: Right coronary artery; SPT: Spasm provocation test

The patient was admitted for coronary angiography (CAG) and spasm provocation test (SPT) to investigate the cause of chest pain at rest or during stress, which was observed with sublingual NTG administration. Acetylcholine (ACh) administration is generally contraindicated in bronchial asthma, but asthma attacks were suppressed under the medications. The usefulness and risks of the tests were explained to the patient, and her consent was obtained. The CAG revealed no significant stenosis, and the SPT using ACh was continued. The usual chest pain appeared when 100 μg of ACh was administered into the left coronary artery, and an electrocardiogram revealed negative T waves at V2-V4 (Figure 1). CAG detected a diffuse spasm in the left anterior descending coronary artery (LAD) (Figure 2). The coronary spasm resolved spontaneously; however, the catheter for the right coronary artery (RCA) could not be changed owing to the radial artery spasm (RAS). Thus, 0.3 mg of NTG was administered intracoronary, which gradually improved RAS, allowing the catheter for RCA to be changed. Ten minutes after NTG administration, a next SPT was performed in the RCA to evaluate the possibility of multivessel spasms. ACh at 50 μg and 80 μg were administered into the RCA, but symptoms, such as chest pain or coronary artery spasms, were not observed (Figure 2). After the SPT, coronary microvascular function using a pressure wire in response to peripheral infusion of adenosine triphosphate was evaluated both in the LAD and RCA. Coronary flow reserve and index of coronary microcirculatory resistance were within normal limits with LAD of 2.4 and 24.5 and RCA of 2.3 and 19.0, respectively. Based on the results of SPT and coronary microvascular function tests, the patient was diagnosed with VSA. The patient was started on oral diltiazem at 100 mg as treatment and was discharged the next day without SPT complications.

Thereafter, treatment was continued on an outpatient basis. The dose of diltiazem was increased to 200 mg bid as the chest pain on light exertion persisted, and oral nicorandil at 15 mg bid was started and is being followed. The duration and frequency of chest symptoms have improved since then, probably because she was reassigned to a less stressful workplace.

## Discussion

Herein, we report a case of a female patient in her 20s diagnosed with VSA. We suspected VSA based on symptoms, such as resting chest strangulation that occurred primarily at rest or during stress and resolved with NTG administration and diagnosed the patient with VSA based on SPT results. In general, VSA mostly occurs after middle age, and the number of patients with VSA tends to increase with age, with a relatively rare onset at a young age (<40 years) [7]. Recently, Sueda et al. [10] reported 18 cases of juvenile VSA in patients <20 years of age, showing that multivessel spasm is more common in juveniles and is as severe as refractory VSA. Thus, because VSA is also present in younger patients, it is important to keep VSA as a differential disease even in young patients who are aware of chest pain.

Various factors are believed to be involved in VSA development [7,12-14], but the risk factors for juvenile VSA are unclear. Through this case study, we discuss the causes and risk factors for VSA in young adults. In this study, FMD was within the normal range at 8.7%, while NID was abnormally high at 27.1%, which may indicate relative vascular endothelial dysfunction. RHI-PAT was also abnormally low. Undoubtedly, vascular dysfunction, including endothelial function, was present. First, smoking is conventionally considered an important factor in VSA development [13], even in females [12]. Among female patients with VSA in Japan, the history of smoking is considerably higher in patients <50 years [9] or 60 years [11], and smoking is reported as a risk factor for coronary spasms in females with VSA. The patient in the present case had a 6-month smoking history, with minimal dose and duration; however, smoking may contribute to VSA development. Second, we discussed the complication of bronchial asthma as a risk factor for VSA. Bronchial asthma complications may predispose patients to coronary spasms [14]. Chronic inflammation can cause vascular endothelial dysfunction, which is considered one of the pathophysiologic mechanisms of coronary spasm [4]. Sueda et al. [15] reported that coronary spasm associated with bronchial asthma is more frequently multivessel spasm that involves the LAD and that bronchial asthma may be associated with coronary spasm severity or activity. This patient had been treated for bronchial asthma for >1 year. Elevated inflammatory markers on blood tests were not present during the outpatient visit, but bronchial asthma may contribute to the development of coronary spasms in the present case. However, it is also true that ACh is contraindicated for use in bronchial asthma, and the above paper [15] may have biased subjects. Large registries and/or prospective studies will be needed to clarify the relationship between bronchial asthma and coronary spasms in the future. Third, stress may contribute to coronary spasm development by affecting the sympathetic/parasympathetic balance [4,16]. This patient had an onset of anginal attacks after work stress, with an improved frequency after reassignment at work, which may have contributed to the onset and pathogenesis of VSA. Other factors, such as female hormones [8] and heredity [4,17], may be involved in the pathogenesis of coronary spasms in young or female patients. However, this case showed no association between the menstrual cycle and the occurrence of anginal attacks, as well as no clear family history of coronary artery disease; thus, these factors seem unlikely to be involved.

The SPT was performed by radial artery puncture in this case, and NTG had to be used due to RASs during the SPT. Sueda et al. reported no RAS during the SPT performed by radial artery puncture [18]. However, studies reported that RAS is more likely to occur when NTG administration cannot be used immediately after radial artery puncture [19], and other studies reported an association between RAS and coronary spasm [20]. The possibility of RAS should be considered when conducting an SPT via radial artery puncture, as in this case. The use of a catheter that is shared by both the left and right coronary arteries may be appropriate for the SPT.

## Conclusions

VSA occurs most frequently in middle-aged and older adults, especially after menopause in women. Although rare, it can also occur in young people. The possibility of VSA should be included in the differential diagnosis to identify chest pain in young patients. A detailed interview, including the risk factors, such as smoking history, bronchial asthma, family history, menstrual history, and stress status, should be conducted, and an SPT should be performed if necessary to establish a definitive diagnosis. A firm diagnosis of VSA is expected to lead to lifestyle modification, the administration of appropriate medicines, and improved drug compliance.
